# Diagnostic and Therapeutic Challenges of Diogenes Syndrome – a clinical case

**DOI:** 10.1192/j.eurpsy.2026.11575

**Published:** 2026-07-31

**Authors:** I. M. Marques

**Affiliations:** Department of Psychiatry, Unidade Local de Saúde de Coimbra, Coimbra, Portugal

## Abstract

**Introduction:**

Diogenes Syndrome (DS) is a behavioral disorder typically seen in the elderly, marked by rejection of social norms, progressive isolation and profound self-neglect (Czarnota-Kuczyńska J et al. J Educ Health Sport 2024; 58:210–24).

**Objectives:**

Through a clinical case, we aim to explore DS, with particular emphasis on associated comorbidities and multidisciplinary therapeutic approaches.

**Methods:**

Case description supported by a review of the literature.

**Results:**

An 83-year-old woman, living alone, was referred to Psychiatry due to cognitive decline. The case was reported by neighbors after repeated refusal of medical care, placing herself and others at risk. She presented behavioral disorganization, unsanitary living conditions, neglect of hygiene, poor treatment adherence and refusal of psychosocial interventions. Laboratory tests revealed vitamin deficiencies, thyroid dysfunction and urinary tract infection. She was admitted for stabilization and social assessment. Clinical improvement was achieved with low-dose antipsychotic therapy and management of comorbidities. She was discharged to a residential care facility with stable condition.

**Conclusions:**

DS is a growing social and public health concern. This case highlights the complex interplay between psychiatric symptoms, medical comorbidities, and social determinants in DS. Detection often relies on neighbors or relatives, as lack of insight leads to refusal of care, complicating intervention. This subject is particularly relevant due to the inherent challenges in diagnosis and treatment, compounded by multiple ethical and legal implications (such as access to the home, environmental interventions and the involvement of public health authorities) (Sacchi L et al. BJPsych Open 2021; 7:e43). The fundamental challenge is to reconcile individual autonomy with the responsibility to safeguard both the person concerned and society at large. No standardized pharmacological treatment exists, as DS is not formally recognized as a distinct clinical entity (Proctor C et al. Case Rep Psychiatry 2021; 2021:2810137). Management must be individualized, addressing comorbidities and integrating social services. Continuous professional training, strengthened support networks and community awareness are crucial for effective intervention.

**Disclosure of Interest:**

None Declared

